# Assessment of sleep phase, quality, and quantity: the development and validation of the 3-dimensional sleep scale (3DSS)

**DOI:** 10.1007/s41105-025-00600-0

**Published:** 2025-07-28

**Authors:** Yuuki Matsumoto, Naohisa Uchimura, Tetsuya Ishida, Motohiro Ozone, Kunitaka Kumadaki, Ayako Hino, Yuichiro Otsuka, Osamu Itani, Yoshitaka Kaneita

**Affiliations:** 1 Department of Neuropsychiatry, Kurume University School of Medicine, Kurume, Japan; 2 Division of Public Health, Department of Social Medicine, Nihon University School of Medicine, Tokyo, Japan; 3 Kurume University School of Nursing, Kurume, Japan; 4 The First Department of Internal Medicine, University of Occupational and Environmental Health, Kitakyushu, Japan; 5 Department of Mental Health, Institute of Industrial Ecological Sciences, University of Occupational and Environmental Health, Kitakyushu, Japan; 6 International University of Health and Welfare, Chiba, Japan

**Keywords:** 3-dimensional sleep scale (3DSS), Reliability and validity, Sleep quality, Sleep quantity, Sleep phase

## Abstract

The 3-Dimensional Sleep Scale (3DSS) is used to assess sleep quality, quantity, and phase. However, its reliability and validity were established using classical methods and it has a small validation sample size. We aimed to increase the sample size and update the statistical methods to validate the reliability and validity of the 3DSS. We conducted a web-based survey of approximately 3000 day-shift workers at four companies in Tokyo, Japan, between 2021 and 2022. The 15 questions in the 3DSS were tested for reliability and validity according to the COSMIN checklist. The maximum likelihood method and promax rotation were selected for exploratory factor analysis. Reliability was tested by calculating the intraclass correlation coefficient and McDonald’s ω reliability coefficient. Convergent and discriminant validity were verified using multi-trait multi-method analysis. Cutoff values were verified using receiver operating characteristic curves and area under the curve (AUC). Existing measures used to validate the validity and cutoff values included the Athens Insomnia Scale, social jetlag, weekday sleep duration, and holiday waking time. Overall 2605 participants were included in the analysis after excluding non-responders, non-consenters, and absent employees. Exploratory factor analysis confirmed a three-factor structure for sleep phase, quality, and quantity. For convergent and discriminant validity, ideal correlations were observed for all subscales. The AUCs for sleep phase, sleep quality, and sleep quantity were 0.747, 0.812, and 0.819, respectively. The 3DSS offers sufficient reliability and validity as a sleep assessment scale. An English version of this scale should be developed in the future.

**Supplementary Information:**

The online version contains supplementary material available at 10.1007/s41105-025-00600-0.

## Introduction

The various sleep scales used in sleep research have unique characteristics. For example, the Pittsburgh Sleep Questionnaire (PSQI) includes sleep quality issues such as difficulty falling asleep, mid-wake, and sleep efficiency, as well as quantity issues such as sleep duration and daytime sleepiness [1–3]. The PSQI includes questions on snoring, pain, and sleeping pills, it is often used in sleep studies targeting patients with certain illnesses and older individuals [6–8]. In contrast, the Athens Insomnia Scale (AIS) contains only simple questions and can be evaluated with a crude score [9, 10]. Nevertheless, both scales cannot assess issues related to sleep phases, such as social jetlag. Social jetlag, which refers to the mismatch between social time and biological time [11], is associated with a variety of physical and mental health problems [12–16].

In 2014, we developed the 3-Dimensional Sleep Scale (3DSS) for Japanese day-shift workers [17, 18]. The 3DSS is a novel scale that consists of only 15 items and allows for simple evaluation not only of sleep quality and sleep quantity, as assessed by conventional sleep scales, but also of sleep phase. When developing this scale, input from sleep specialists, psychiatrists, industrial physicians, and clinical psychologists was used to establish its content validity and verify its reliability. However, the original study had limitations. First, the sample size was small. Second, although the principal factor method used for exploratory factor analysis is suitable for small sample sizes, it has limitations such as lower accuracy in estimating factor loadings and the inability to test model fit. Third, the concept of social jetlag had not yet become widely recognized at the time, and there were no appropriate indicators available to examine the convergent validity of the sleep phase score. Therefore, this study aimed to update the analytical methods and retest the reliability and validity of the 3DSS using a larger sample size.

## Methods

### Study design, participants, and ethical considerations

This study used an online questionnaire administered from October 2021 to December 2022. The participants were 3609 day-shift workers employed by four companies in Tokyo who cooperated with the survey. Because the population of Tokyo is approximately 10 million, we considered a sample size of at least 1067 persons sufficient, assuming a 95% confidence level, a 3% margin of error, and a population ratio of 0.5. All workers at the four companies in this study worked a day shift.

The purpose and procedures of the study were explained to the employees. Participation was voluntary, and we explained that no disadvantages would arise from not participating in the study and that the data obtained would be used exclusively for the study. First, a survey form was created online, and the staff in charge of each company sent its URL to all employees via e-mail with a request for responses. Consent for participation was obtained through a web-based interface and received electronically; those who disagreed were barred from accessing and responding to any research items. Identifying information was anonymized using a substitute employee ID number, and the participants were informed that their responses would be deleted if they chose to withdraw consent after answering the study.

### Constitutive concepts and items of the 3DSS

The 3DSS was designed for use by Japanese day workers [17]. It comprises three categories (sleep phase, sleep quality, and sleep quantity), each with 5 items (for a total of 15 items). All items were discussed with sleep specialists, psychiatrists, occupational physicians, and clinical psychologists to ensure sufficient content validity. The constitutive concepts of each subscale and scoring methods are described in Supplementary material 1.

### Response bias, construct validity, reliability, and reproducibility

The 15 questions of the 3DSS were tested for reliability and validity according to the COSMIN checklist, an international standard for scale development [19–21]. We analyzed the ceiling effect, floor effect, skewness, and kurtosis to check for response bias. For construct validity, we performed exploratory factor analysis using the maximum likelihood method and promax rotation. Reliability was tested by calculating the intraclass correlation coefficient and McDonald’s ω reliability coefficient [22]. To verify reproducibility, participants were asked to respond again within 2 weeks to the 15 items of the 3DSS, and the intraclass correlation coefficient between the first and second responses was calculated.

### Indicators for convergent and discriminant validity

We tested convergent and discriminant validity using a multitrait-multimethod matrix that correlated with existing indicators. The existing indicators used were holiday wake-up time, social jetlag, the AIS [4, 5], and weekday sleep duration.

Holiday wake-up times and social jetlag were used as phase indicators. The sleep phase subscale of the 3DSS includes constructs related to chronotype and sleep rhythm regularity. The holiday wake-up time is a good indicator of chronotype because it reflects an individual's true wake-up time, unaffected by social constraints, and social jetlag is obtained from the difference between the mid-sleep time on weekdays and mid-sleep time on weekends; the extent of social jetlag is larger for those whose sleeping and wake-up times change between weekdays and holidays [23–25].

The sleep quality subscale of the 3DSS includes constructs related to nocturnal symptoms associated with insomnia disorders, such as difficulty falling asleep, awakening in the middle of the night, and early morning awakening. Okajima et al. reported that the AIS has a two-factor structure with Q1–5 and Q6–8 [5]. Of these, Q1–5 of the AIS are good indicators of sleep quality, including difficulty falling asleep and awakening during the middle of the night. We therefore used the total AIS Q1–5 score to assess sleep quality.

The sleep quantity on the 3DSS subscale includes not only sleep duration but also daytime symptoms such as daytime sleepiness, fatigue, and dozing (unintentional sleep) caused by lack of sleep. Q6–8 of the AIS are good indicators of sleep quantity as they focus on daytime symptoms. The total score of weekday sleep duration and Q6–8 of the AIS were therefore used as measures of quantity.

### Hypothesis for convergent and discriminant validity

First, we hypothesized convergent validity; we expected the sleep phase score to be strongly correlated (− 0.8 ≤ *r* ≤  − 0.5) with the time of waking on holidays because the sleep phase score includes items directly related to waking time. In contrast, since the sleep phase score does not include items directly asking about social jetlag, we expected a moderate correlation (− 0.5 ≤ *r* ≤  − 0.3) between social jetlag and the sleep phase score. Quality 1–3 in the sleep quality score of the 3DSS is quite close to Q1–3 of the AIS because both are based on nocturnal insomnia symptoms. Therefore, we expected a strong correlation (− 0.8 ≤ *r* ≤  − 0.5). Quantity 3–5 included in the sleep quantity score assessed insomnia symptoms upon awakening, as did Q6–8 in the AIS. Therefore, we expected a strong correlation (− 0.8 ≤ *r* ≤  − 0.5). Since only one item, quantity 1, contained a question about sleep duration on weekdays, we expected a moderate correlation (0.3 ≤ *r* ≤ 0.5).

Next, hypotheses were set up for discriminant validity: the sleep phase scores were predicted to be largely uncorrelated (− 0.3 ≤ *r* ≤ 0.3) with AIS Q1–5, Q6–8, and weekday sleep duration. The sleep quality scores predicted little correlation (− 0.3 ≤ *r* ≤ 0.3) with holiday wake-up time, social jetlag, and weekday sleep duration. Q6–8 of the AIS contains items related to mood, which may have some correlation with nocturnal symptoms of insomnia. Therefore, we predicted that a moderate correlation (− 0.5 ≤ *r* ≤  − 0.3) between the sleep quality score of the 3DSS and Q6–8 of the AIS would be observed, which would be weaker than the correlation with Q1–5 of the AIS. The sleep quantity score was predicted to show minimal correlation (− 0.3 ≤ *r* ≤ 0.3) with holiday waking time and social jetlag. For AIS Q1–5, Q4 contained items related to sleep duration. Therefore, we predicted that the sleep quantity score of the 3DSS would have a moderate correlation (− 0.5 ≤ *r* ≤  − 0.3) with Q1–5 of the AIS and a weaker correlation with Q6–8 of the AIS.

### Validation of cutoff values

We decided to use social jetlag and AIS, which were used during convergent validation, as indicators to validate the cutoff values. Social jetlag is the reported risk of health problems when there is more than 1 h [12, 13]. Therefore, we used “social jetlag ≥ 1 h” as the outcome when validating the cutoff value for the phase score. An “AIS score of 6 points or more”, the threshold for a suspected sleep disorder, was set as the outcome when validating the cutoff values of the 3DSS sleep quality score and sleep quantity score. The area under the curve (AUC) was determined from the receiver operating characteristic (ROC) curve, and the maximum Youden’s index score was searched to confirm whether there were any major discrepancies with the current cutoff values.

### Statistical analysis

We employed the maximum likelihood method with Promax rotation for exploratory factor analysis. Factor loading cutoff values are often set between 0.3 and 0.4 [17, 26–28]. In the present study, we used a large sample size and employed the maximum likelihood method, which provides more precise estimates of factor loadings; therefore, we set the cutoff value at 0.3. Spearman’s correlation coefficient was used for the correlation analysis. The scale’s reliability was assessed using the McDonald’s ω coefficient [22]. For the reliability value, 0.65 or higher was considered acceptable, based on criteria frequently used in previous research [29–31]. All analyses were performed using IBM SPSS Version 28 for Windows. A two-sided p-value < 0.05 was considered statistically significant.

## Results

### Participant characteristics

Responses were obtained from 2651 participants. Among these, 35 did not consent to participate in the study, and 11 had been on leave within the past month, leaving 2605 (1718 men and 887 women; mean age 42.2 years, standard deviation 11.7) individuals included in the analysis. The valid response rate was 72.2%. The basic characteristics of the analyzed participants are presented in Supplementary material 2.

### Response polarization analysis

Table 1 shows the mean (M), standard deviation (SD), ceiling effect (M + SD, upper limit 3), floor effect (M-SD, lower limit 0), kurtosis, and skewness for each item. Ceiling and floor effects were assessed for each item. Although a small ceiling effect was observed for Quality 3 (3.10), neither kurtosis nor skewness exceeded 1.

**Table 1:** Characteristics of missing and score distributions for each item

	Phase 1	Phase 2	Phase 3	Phase 4	Phase 5	Quality 1	Quality 2	Quality 3	Quality 4	Quality 5	Quantity 1	Quantity 2	Quantity 3	Quantity 4	Quantity 5
Mean	1.88	1.61	1.67	1.33	1.49	1.97	2.02	2.32	1.58	2.10	1.52	1.37	1.59	1.67	2.10
SD	0.89	0.96	1.09	1.01	1.14	0.94	0.97	0.78	0.90	0.88	0.96	0.94	0.87	0.86	0.78
Ceiling effect	2.77	2.57	2.76	2.34	2.64	2.91	2.99	3.10	2.49	2.98	2.49	2.31	2.46	2.53	2.88
Floor effect	0.99	0.65	0.59	0.32	0.35	1.03	1.05	1.54	0.68	1.22	0.56	0.43	0.72	0.82	1.31
Skewness	− 0.40	− 0.12	− 0.23	0.20	0.01	− 0.56	− 0.60	− 0.86	− 0.05	− 0.67	− 0.04	0.12	− 0.08	− 0.13	− 0.53
Kurtosis	− 0.59	− 0.93	− 1.24	− 1.05	− 1.41	− 0.63	− 0.75	− 0.10	− 0.79	− 0.39	− 0.95	− 0.88	− 0.67	− 0.64	− 0.26

### Exploratory factor analysis

**Fig. 1:**
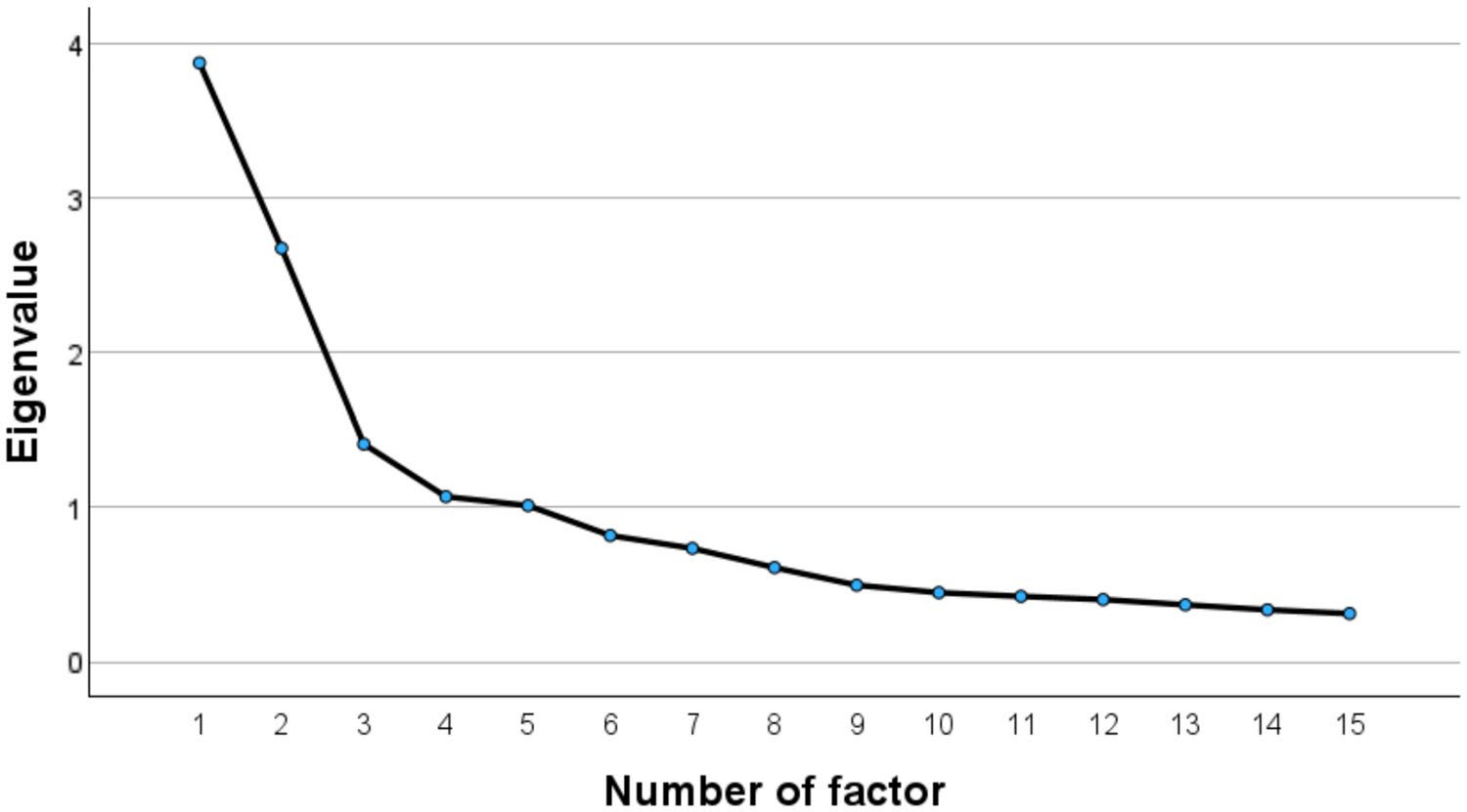
Scree plot of factors

Exploratory factor analysis was performed on 15 items. Referring to the scree plot (Fig. 1) and the scale construct, the number of factors was set to three. Table 2 presents the results of the factor analysis. The Kaiser–Meyer–Olkin measure of sampling was 0.790, Bartlett’s sphericity test was significant (p < 0.001), and the cumulative contribution rate was 53.1%. The goodness-of-fit test results were statistically significant (p < 0.001). Factor loadings above 0.3 were observed for all items. The first factor consisted entirely of quality items (quality 1–5). The second factor consisted entirely of phase items (phase 1–5). The third factor consisted entirely of quantity items (quantity 1–5). Although quality 4 had factor loadings of over 0.3 not only for both the first factor and third factor, the factor loading on the 1st factor was clearly larger. In other words, it was regarded as belonging to the first factor. As all questionnaire items had factor loadings ≥ 0.3 for one factor, the exploratory factor analysis was considered complete.

**Table 2:** Factor loadings and communality based on exploratory factor analysis

Items	Factor		
	1st	2nd	3rd
Quality 2	0.725	0,159	− 0.133
Quality 5	0.678	− 0.077	0.085
Quality 3	0.637	0.232	− 0.040
Quality 1	0.630	− 0.202	− 0.090
Quality 4	0.577	0.025	0.311
Phase 2	0.095	0.737	− 0.049
Phase 1	0.033	0.664	− 0.011
Phase 5	− 0.056	0.587	0.231
Phase 4	− 0.109	0.571	− 0.113
Phase 3	− 0.074	0.358	0.035
Quantity 1	− 0.273	0.190	0.806
Quantity 2	0.090	0.045	0.771
Quantity 3	0.277	− 0.198	0.414
Quantity 4	0.119	− 0.150	0.407
Quantity 5	0.077	− 0.036	0.311

### Reliability coefficients, item-total correlation analysis, and intraclass correlation coefficient

The reliability coefficients, item-total correlation, and intraclass correlation coefficients for each subscale are presented in Table 3. The McDonald’s ω coefficient for sleep phase, sleep quality, and sleep quantity were 0.690, 0.786, and 0.710, respectively. Therefore, all reliability coefficients were above the cutoff value of 0.65. For item-total correlation, all items had significant correlations exceeding 0.3, and no items had reliability coefficients that increased by more than 0.1 when excluded. The second dataset of responses was obtained from 402 participants to verify reproducibility. Intraclass correlation coefficients for reproducibility, close to or greater than 0.7, were observed for all items.

**Table 3:** Reliability, item-total correlation coefficient, and correlation coefficients for reproducibility in the three subscales

	Reliability coefficient	Item	Item–total correlation	Reliability coefficient when the item is excluded	Intraclass correlation coefficient for reproducibility (n = 402)
Sleep phase	ω = 0.690	Phase 1	0.485	0.658	0.686
		Phase 2	0.557	0.629	0.828
		Phase 3	0.317	0.691	0.887
		Phase 4	0.530	0.600	0.891
		Phase 5	0.450	0.666	0.908
Sleep quality	ω = 0.786	Quality 1	0.507	0.766	0.828
		Quality 2	0.567	0.758	0.836
		Quality 3	0.544	0.757	0.807
		Quality 4	0.597	0.730	0.816
		Quality 5	0.633	0.730	0.811
Sleep quantity	ω = 0.710	Quantity 1	0.401	0.706	0.862
		Quantity 2	0.591	0.641	0.780
		Quantity 3	0.507	0.628	0.704
		Quantity 4	0.546	0.679	0.752
		Quantity 5	0.398	0.708	0.758

*Note* All correlation coefficients (Spearman’s correlation coefficients) were significant (p < 0.05)

### Hypothesis verification for convergent validity and discriminant validity

The convergent and discriminant validity results, based on the multi-trait multi-method analysis, are presented in Table 4. For convergent and discriminant validity, the hypothesized ideal correlation was confirmed for all subscales.

**Table 4:** Convergent validity (bold italic) and discriminant validity (italic)

		Indicators				
		Sleep phase		Sleep quality	Sleep quantity	
		Wake up time on day off	Social jetlag	AIS quality score	AIS quantity score	Sleep duration
3DSS	Sleep phase	− 0.735^*^ (− 0.8 ∼ − 0.5)	− 0.484^*^ (− 0.5 **∼** − 0.3)	− 0.041^*^ (− 0.3 **∼** 0.3)	− 0.174^*^ (− 0.3 **∼** 0.3)	*0.003* (− 0.3 **∼** 0.3)
	Sleep quality	0.081^*^ (− 0.3 **∼** 0.3)	0.040^*^ (− 0.3 **∼** 0.3)	− 0.753^*^ (− 0.8 ∼ − 0.5)	− 0.340^*^ (− 0.5 **∼** − 0.3)	* − 0.021* (− 0.3 **∼** 0.3)
	Sleep quantity	− 0.147^*^ (− 0.3 **∼** 0.3)	− 0.130^*^ (− 0.3 **∼** 0.3)	− 0.472^*^ (− 0.5 **∼** − 0.3)	− 0.675^*^ (− 0.8 ∼ − 0.5)	0.336^*^ (0.3 **∼** 0.5)

*Note* The hypotheses are given in parentheses

^*^ Spearman’s correlation coefficient is significant (p < 0.05)

### Confirmation of cutoff values

**Fig. 2:**
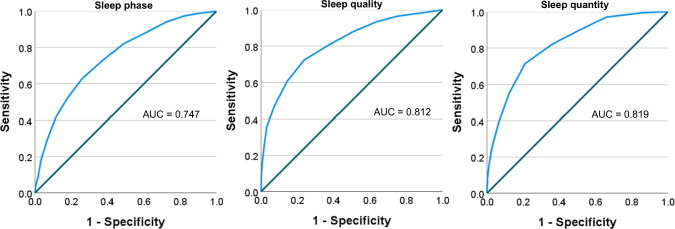
Receiver operating characteristic curve of each subscale scores

For all subscales, the score ranged from 0 to 15. The M ± SD scores for sleep phase, quality, and quantity were 8.0 ± 3.5, 10.0 ± 3.3, and 8.3 ± 3.1, respectively. The ROC curve for these three scores are shown in Fig. 2; the AUC were 0.747, 0.812, and 0.819. The sensitivity, specificity, and (sensitivity + specificity − 1) for each point are listed in Table 5. For sleep phase score, the maximum Youden index was 7.5, while the current cutoff was 8.5. For sleep quality score, the maximum Youden index was 9.5, and the current cutoff was 10.5. For sleep quantity score, the maximum Youden’s index was 7.5, while the current cutoff was 8.5.

**Table 5:** Sensitivity and specificity of each subscale score

Cutoff	Sensitivity			Specificity			Sensitivity + Specificity − 1		
	Sleep phase	Sleep quality	Sleep quantity	Sleep phase	Sleep quality	Sleep quantity	Sleep phase	Sleep quality	Sleep quantity
0.5	0.012	0.003	0.013	0.998	0.999	0.999	0.010	0.002	0.013
1.5	0.040	0.018	0.032	0.995	0.999	0.999	0.035	0.016	0.031
2.5	0.099	0.057	0.082	0.980	0.998	0.997	0.079	0.055	0.079
3.5	0.178	0.097	0.142	0.967	0.998	0.990	0.145	0.094	0.132
4.5	0.295	0.157	0.252	0.931	0.993	0.972	0.226	0.150	0.224
5.5	0.415	0.244	0.389	0.885	0.983	0.935	0.300	0.228	0.324
6.5	0.518	0.358	0.552	0.823	0.968	0.876	0.341	0.326	0.428
7.5	0.628	0.472	0.714	0.740	0.925	0.788	0.368^*^	0.398	0.503^*^
8.5	0.728	0.607	0.821	0.627	0.855	0.639	0.355^†^	0.462	0.460^†^
9.5	0.821	0.723	0.901	0.512	0.763	0.486	0.333	0.485^*^	0.387
10.5	0.886	0.807	0.971	0.382	0.625	0.340	0.268	0.432^†^	0.311
11.5	0.940	0.879	0.985	0.278	0.495	0.228	0.218	0.375	0.213
12.5	0.973	0.934	0.995	0.181	0.366	0.143	0.154	0.300	0.138
13.5	0.987	0.965	0.998	0.106	0.251	0.081	0.093	0.216	0.079
14.5	0.994	0.981	1.000	0.049	0.132	0.031	0.043	0.114	0.031

^*^ Maximum Youden index

^†^ Current cutoff value’s Youden index

## Discussion

In this study, the new methods (i.e., maximum likelihood method, McDonald’s ω reliability coefficient, and reproducibility) were used with a larger sample size than that in our previous study of 3DSS, which resulted reliability and validity in higher accuracy. Furthermore, using social jetlag and sleep duration as indicators for testing convergent and discriminant validity enhances the generalizability of our results.

### Response bias and construct validity

In the response bias analysis of the 15 items, only Quality 3 had a ceiling effect. However, because it was slight and no kurtosis or skewness exceeded |1|, this result alone did not warrant exclusion. Regarding structural validity, exploratory factor analysis based on the maximum likelihood method indicated a three-factor structure with five items each, as expected. Quality 4, although factor loadings exceeding 0.3 were observed for both factors, was not excluded because the factor loadings for the first factor were clearly higher than those for the third factor. The decision not to exclude Quality 4 was also considered appropriate, considering the reliability coefficient when the item was excluded and the clinical importance of this item, which asks about sleep disturbance.

### Reliability and reproducibility

Reliability coefficients above or close to 0.7 were observed for all subscales, indicating that the scales were sufficiently reliable. The results of the intraclass correlation coefficient, which examined reproducibility, showed that most items had strong correlations exceeding 0.75, indicating high reproducibility. The results of Phases 1 and Quantity 3, which were approximately 0.7, were not low enough to be ruled out as not reproducible, and the results of other analyses were taken into consideration.

### Convergent validity and discriminant validity

Regarding convergent validity, all subscales were correlated with relevant existing indicators to the assumed degree and direction, indicating their validity. Regarding discriminant validity, all subscales showed no more correlations than expected, with existing indicators measuring different factors, indicating their validity. In particular, the sleep phase score showed almost no correlation with existing indicators of sleep quality and quantity, suggesting that it is completely discriminative and that it showed a correlation with social jetlag, providing novel insights into factors not measurable by other sleep scales, such as the PSQI and AIS. The AIS evaluates the total score of Q1–8 because it is used to evaluate insomnia disorders. The diagnostic criteria for insomnia disorders include daytime symptoms in addition to nighttime symptoms [32]. Q1–5 in the AIS are items related to nighttime symptoms, while Q6–8 are items related to daytime symptoms. Consequently, although they were extracted separately as factors, both are found in insomnia; therefore, some correlation is possible.

### Cutoff values

The ROC curves, using social jetlag and AIS as outcomes, showed that the AUC area for all subscales exceeded 0.7. These results indicate that screening based on 3DSS is comparable to the accuracy in the case of screening based on social jetlag ≥ 1 h or AIS score ≥ 6. Regarding the cutoff value, the score that maximized Youden’s index was one point lower than the current value, which itself was one point lower than the current sensitivity. Since Japan has a lower sleep condition compared to other countries [33], the current setting of “a cutoff value that captures many individuals (i.e., a cutoff value with high sensitivity)” would be acceptable. However, in situations where there is a shortage of personnel that limits the ability to respond to positive cases, or when the target population is expected to have a high level of health and a low pre-test probability, it may be more practical to lower the cutoff score by one point.

### Limitations and future prospects

This study had some limitations. First, because all participants were workers, its reliability and validity cannot be guaranteed when applied to those who are not working. Second, the number of participants were not large in the study when verifying reproducibility. However, this number may be considered sufficient considering the practical challenge of conducting the same survey twice with busy workers. Third, there may be some degree of bias in the present study, as the sample included a slightly higher proportion of male participants and was drawn exclusively from Tokyo. Last, 3DSS created in Japanese for Japanese participants. In the future, it will be necessary to increase the proportion of female participants and include workers from regional cities in order to conduct confirmatory factor analysis under more diverse conditions. Furthermore, since sleep phase issues, as represented by social jetlag, are gaining attention not only in Japan but also in other countries, it is necessary to develop an English version of the 3DSS that can be easily adapted into various languages. In particular, as Japan is known to have shorter sleep duration compared to other countries, it will be necessary to rigorously re-evaluate the reliability and validity of the 3DSS when applying it in different cultural or national contexts.

## Conclusions

In this study, the reliability and validity of the 3DSS developed in 2014 were tested using a new sample population and methodology. The results demonstrated adequate reliability, structural validity, convergent validity, and discriminant validity for use with day shift workers. The 3DSS is the world’s first scale to evaluate sleep phase, quality, and quantity on a single scale; the development of an English version is warranted.

## Supplementary Information

Below is the link to the electronic supplementary material.

Supplementary file1 (DOCX 21 kb)
