# Supplementary material for: Assessment of sleep phase, quality, and quantity: the development and validation of the 3-dimensional sleep scale (3DSS)
Source: Sleep Biol Rhythms. 2025 Jul 28;24(1):29–37. doi: 10.1007/s41105-025-00600-0 (PMC12804515; doi:10.1007/s41105-025-00600-0)
Supplement: Supplementary file 1 — Supplementary file1 (DOCX 21 kb) [file 41105_2025_600_MOESM1_ESM.docx]

**Supplementary material 1.** Constitutive concepts of the subscales in 3DSS

| **Subscales** | **Summary and items of constitutive concepts** | |
| --- | --- | --- |
| **Sleep Phase** | This subscale assesses sleep phase disturbances by scoring whether sleeping and waking times are irregular or regressive. Thus, the Sleep Phase score reflects the regularity and chronotype of habitual sleep rhythms. | |
|  | Phase 1 | I go to bed at a fixed, regular time on weekdays and weekends. |
|  | Phase 2 | I wake up at a fixed, regular time on weekdays and weekends. |
|  | Phase 3 | I have a well-balanced breakfast every day. |
|  | Phase 4 | I am a morning person rather than an evening person. |
|  | Phase 5 | What time do you wake up on weekdays? |
| **Sleep Quality** | This subscale assesses sleep quality by scoring the intensity of nocturnal symptoms of insomnia, such as sleep latency, sleep maintenance, and feeling of sound sleep. Thus, the Sleep Quality score reflects subjective satisfaction and sleep efficiency, i.e., the actual sleep time as a percentage of total bedtime. | |
|  | Quality 1 | It takes me more than 30 min to fall asleep. |
|  | Quality 2 | I wake up more than twice a night. |
|  | Quality 3 | I wake up earlier than usual (≥2 h) and cannot fall asleep again. |
|  | Quality 4 | I do not feel that I have a sound sleep. |
|  | Quality 5 | I worry that I cannot fall asleep. |
| **Sleep Quantity** | This subscale assesses the adequacy of sleep quantity by scoring sleep duration and the intensity of daytime symptoms, such as sleepiness or dozing while awake. Thus, the Sleep Quantity score reflects whether the amount of sleep is sufficient for an individual. | |
|  | Quantity 1 | I sleep for less than 6 h on weekdays. |
|  | Quantity 2 | I cannot get enough sleep even though I want to. |
|  | Quantity 3 | I have sleepiness and/or fatigue when I wake up. |
|  | Quantity 4 | I feel sleepy not only in the afternoon but also in the morning and/or evening. |
|  | Quantity 5 | I doze off when I am supposed to stay awake. |
| **Opening text**  Please select the answer that applies to your daily sleep in the past month.  **Answers**  (Except for phase 5): (1) always, (2) often, (3) rarely, and (4) never.  (For Phase 5): (1) about 6:00 a.m. or earlier, (2) about 6:30 a.m., (3) about 7:00 a.m., and (4) later than 7:00 a.m.  **Scoring**  (phase 1–5): Answers of (1)–(4) were given scores of 3–0.  (quality 1–5 and quantity 1–5): Answers of (1)–(4) were given scores of 0–3.  The score for each subscale ranged from 0 to 15, with higher scores indicating better sleep conditions.  **Cutoff values (poor/good)**  sleep phase score 8/9; sleep quality score, 10/11; and sleep quantity score, 8/9.  **Note**  “Poor sleep phase” indicates irregular and delayed sleep rhythm; “poor sleep quality” indicates low sleep efficiency with symptoms of nocturnal insomnia; and “poor sleep quantity” indicates the presence of daytime arousal disorders associated with insufficient sleep duration. | | |

**Supplementary material 2.** Basic characteristics of the analyzed participants

|  | **n** | **%** |
| --- | --- | --- |
| **total** | 2,605 | 100 |
| **age** |  |  |
| ≤ 29 | 514 | 19.7 |
| 30-39 | 573 | 22.0 |
| 40-49 | 651 | 25.0 |
| 50-59 | 719 | 27.6 |
| ≥ 60 | 148 | 5.7 |
| **sex** |  |  |
| men | 1718 | 66.0 |
| women | 887 | 34.0 |
| **occupations** |  |  |
| sales or marketing | 910 | 34.9 |
| technical or engineering roles | 610 | 23.4 |
| administrative roles | 504 | 19.3 |
| others | 581 | 22.3 |
